# Single versus double symphyseal plating in management of tile C1-2 and C1-3 pelvic ring injuries: a randomized controlled trial

**DOI:** 10.1186/s12893-025-02936-3

**Published:** 2025-05-09

**Authors:** Islam Sayed Moussa, Ibrahim Mahmoud Abdelmonem, Amr Mohammed Nagy

**Affiliations:** Department of Orthopedics and Traumatology, Faculty of Medicine, Ain Shams University, 56 Ramses Street, Abbasia, Cairo, 11522 Egypt

**Keywords:** Anterior ring fixation, Matta & Tornetta score, Majeed pelvic score, Symphyseal plating, Tile C1-2 & C1-3 injuries, Vertical shear injuries

## Abstract

**Background:**

Single superior symphyseal plating is the most effective method for managing vertically unstable Tile C1-2 and C1-3 pelvic ring injuries. However, high rates of implant failure were more frequently observed in obese patients (body mass index < 30). The study aimed to determine the potential reduction in implant failure rates by adding an anterior symphyseal plate in obese patients (class I).

**Methods:**

The study was designed as a prospective, randomized controlled trial with a single-blind methodology, conducted at a level 1 trauma center. The study involved 36 patients with Tile C1-2 and C1-3 injuries, and class I obesity between February 2022 and May 2023. All cases had posterior and anterior ring fixation, with 18 cases having superior symphyseal plating and 18 cases having additional anterior plating (Groups A and B). The primary outcomes were radiological, functional outcomes, and implant failure rates.

**Results:**

Patients in group A were followed up for an average of 13.39 months, and those in group B for 13.7 months. Group A exhibited a significantly shorter operative time with a mean difference of 30 min (*p* < 0.001), as well as lower reoperation rates (*p* = 0.03). Both groups had similar final clinical and radiological outcomes (*p* = 0.44 and 0.78) and implant failure rates (*p* = 0.18) at the last follow-up.

**Conclusion:**

The authors found that using a single high-quality symphyseal plate effectively addresses symphyseal diastasis in vertically unstable Tile C1-2 and C1-3 pelvic ring injuries among patients with class I obesity. This method lowers morbidity by reducing operation times and minimizing reoperation rates, while the inclusion of an additional anterior plate does not enhance the final radiological and clinical outcomes.

**Level of evidence:**

Therapeutic Level I study.

**Trial registration:**

Ain Shams University’s ethical committee retrospectively registered and approved this trial (FWA 000017585 FMASU R65/2022). It was organized and operated according to the guidelines of the International Council on Harmonization (ICH) in Anesthesiology and the Islamic Organization for Medical Sciences (IOMS). The United States Office for Human Research Protections and the United States Code of Federal Regulations operate under Federal Wide Assurance No. 000017585 (retrospectively registered). Our study was registered at ClinicalTrials.gov with clinical trial number NCT06439108 with clinical trial registry ({05/30/2024}.

**Supplementary Information:**

The online version contains supplementary material available at 10.1186/s12893-025-02936-3.

## Introduction

The management of high-energy vertical shear injuries of the pelvis represents a considerable challenge, even for experienced surgeons specializing in pelvic and acetabular procedures. Initial treatment for such injuries is multifactorial, depending on many factors, including the physiological status, body mass index (BMI), associated visceral and urogenital injuries, and lastly, the injury pattern [1]. Vertically unstable Tile C1-2 and C1-3 pelvic ring injuries are life-threatening and require emergent pelvic binder, resuscitation, and first-aid skeletal traction to restore as much as possible of the vertical instability. Urgent surgical intervention and management of associated urogenital or visceral injuries are crucial after resuscitation [2]. 

Anterior pelvic ring disruption, which accounts for about 50% of pelvic ring injuries, can occur at different sites. Pubic rami fractures are stable and heal better with soft tissue support, while Symphyseal diastasis associated with vertical and rotational instability requires stabilization in most cases [3] for early mobility and less pain.

Once first aid measures for a life-threatening condition have been accomplished, proper planning for definitive fixation should be formulated. To identify injury sites and deformities, pelvic ring imaging, combined with multislice CT scan, is required for pelvic ring stability assessment [4]. Many fixation methods were described for such injuries, including symphyseal plating, external fixation, percutaneous fixation, and recently the anterior subcutaneous internal fixator (INFIX) [5]. There was no clear consensus recommending one fixation method over the other; however, many biomechanical studies supported the use of symphyseal plating for adequate restoration of pelvic ring stability and for being the most rigid fixation method for open book injuries, especially in patients with higher BMI [6]. 

**Fig. 1:**
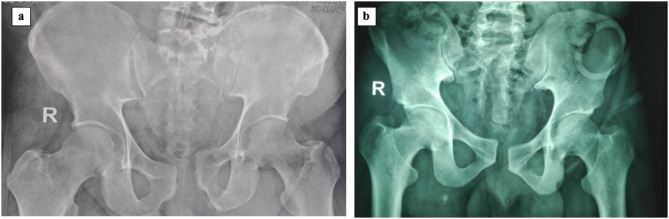
Shows preoperative x-rays that show **a**-Tile C1-2 and **b**- C1-3 injuries, respectively

Posterior percutaneous fixation using iliosacral screws combined with a single superior symphyseal plate is the gold standard for managing Tile C1-2 and C1-3 pelvic ring injuries [7, 8]. However, the question is whether adding anterior symphyseal plate can reduce the incidence of implant failure and recurrent diastasis in obese patients with higher BMI. Several previous studies including the one by Tseng et al. [9] investigated the relationship between implant failure and radiological outcomes following symphyseal plating for Tile C1-2 and C1-3 injuries, however they failed to establish a clear relationship between clinical and radiological outcomes following these interventions. This indicates a knowledge gap in prospectively assessing these outcomes. Therefore, this study will be the first to include these types of outcomes prospectively. The algorithm encountered was whether an additional anterior symphyseal plate was necessary for managing Tile C1-2 and C1-3 pelvic ring injuries with symphyseal diastasis in obese patients (Fig. 1). The researchers aimed to compare the complications’ rate and radiological and functional outcomes between two methods: using a superior symphyseal plate with and without an additional anterior plate. The study hypothesis was that using an additional anterior plate would result in a more rigid fixation and fewer complications. The goal was to achieve these advantages without increasing patient morbidity by using the same incision to apply the additional plate.

### Patients and methods

Following approval from the Hospital Research/Ethics Committee (FWA 000017585 FMASU R65/2022), investigators conducted a randomized clinical trial (RCT) from February 2022 to May 2023, identified by clinical trial number NCT06439108 with clinical trial registry ({05/30/2024}. All patients provided written informed consent. The study included a thorough screening process, comprising a clinical assessment, physical examination, and radiological investigations such as pelvis X-rays (anteroposterior, inlet, and outlet views) and CT scans preoperatively, to enroll patients. The patient’s BMI was calculated using the arm span measurement [10] from the sternal notch to the top of the middle finger to calculate the patients’ height, and the weight was calculated via an estimation formula based on the waist circumference [11]. The investigators meticulously analyzed results alongside the latest pre-traumatic records. With the expertise of two independent radiologists, patients were classified using the Tile classification system, and participants who fulfilled the inclusion criteria were actively recruited in the study. 42 patients aged 18–60 with Tile C1-2 and C1-3 pelvic ring injuries and class I obesity (BMI 30–35) [12] were recruited, excluding those with other associated urogenital or visceral injuries, previous abdominal operations, uncontrolled major medical comorbidities, pubic rami fractures, open fractures or fractures lasting more than two weeks.

**Fig. 2:**
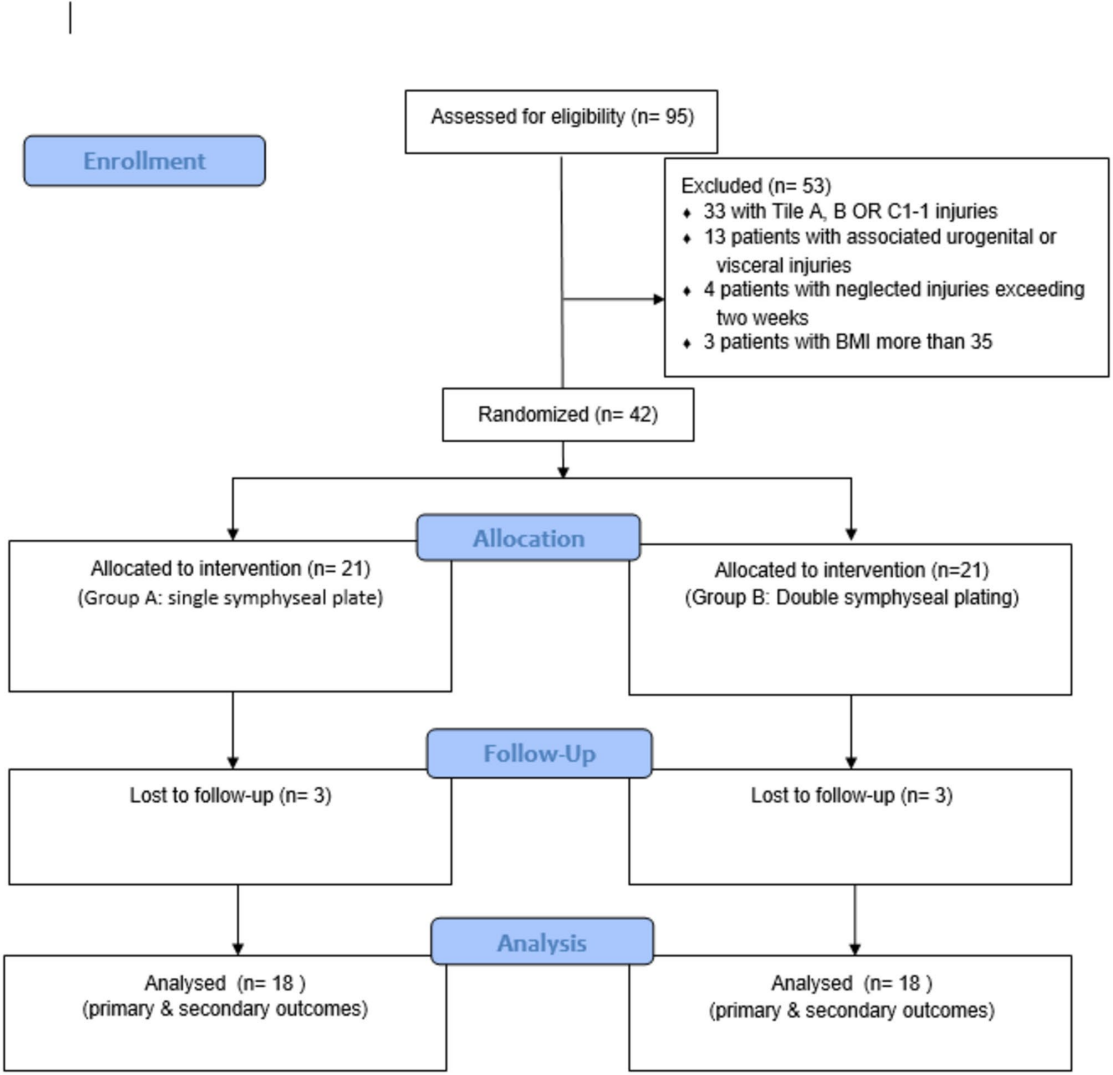
The study group’s consort flow diagram

The authors utilized computerized randomization with a 1:1 allocation of sample numbers in random blocks 2, 4, 6, and 8, with an even distribution between the two groups. To achieve block randomization, cases were assigned randomly within each block. This ensured that each treatment received an equal number of participants. Firstly, a group of participants was randomly selected from the orderings and then assigned to study groups according to the established sequence. In this manner, the procedure of allocation was carried out. A third blinded independent doctor evenly assigned sample numbers to two groups: one group received a single symphyseal plate (Group A), while the other received double symphyseal plating (Group B). Thirty-six patients (86%) in the analysis completed the final follow-up; six cases were lost to follow-up (Fig. 2); two patients died, two were unavailable, and two cases requested withdrawal. The allocation of cases was double-blinded, with both the participants and the outcome assessors unaware of the treatment selection.

Two expert consultant orthopedic surgeons in pelvic ring and acetabular trauma management operated on all the included cases; anterior ring open reduction and fixation were started using a Pfannenstiel incision; restoration of symphyseal vertical and rotational displacement via traction and pelvic reduction clamp. The patients were divided into two groups: Group A included 18 cases (50%) that received a single symphyseal plate (three holes on either side), while Group B included 18 cases (50%) that received double superior symphyseal plate (three holes on either side) and anterior 4-hole plates (two holes on either side), followed by a single SI screw for the management of the posterior ring in all cases. The authors recommended a six-week partial weight-bearing protocol for both groups, requiring them to bear 50% of their body weight with crutches.

The rehabilitation protocol outlined in the study included several key phases. In the second week, hip passive and active-assisted range of motion (ROM) exercises were initiated. By the sixth week, an unassisted weight-bearing program was introduced alongside strengthening exercises for the abductor and quadriceps muscles. The overall goal was to help patients regain full weight-bearing ability and complete range of motion, enabling them to return to work by the end of the third month. After six months and again after one year, patients underwent comprehensive radiological and clinical assessments to evaluate their progress in returning to pre-injury mobility and athletic activities.

The primary outcomes of the study included radiological and clinical results, as well as the rate of implant failure. Secondary outcomes included postoperative complications, average blood loss, average operative time, the necessity for additional surgeries, and intraoperative reduction assessments.

The study involved patients undergoing radiological assessment using Matta & Tornetta principles [13, 14], including pelvis X-rays anteroposterior, inlet, and outlet views at each follow-up visit (1.5, 3, 6 months, and 1-year intervals). At the last follow-up, five criteria were evaluated and analyzed, classifying patients into excellent, good, fair, and poor categories. The Majeed pelvic score [15] was also assessed during this visit, and the patients were classified into excellent, good, fair, and poor categories. Implant failure was defined according to Collinge et al. criteria [16, 17] (plate or screws breakage, lysis around the screw threads, screw cutout, and screw head separation from the implant). The study followed patients for at least 12 months.

The research question of our randomized controlled trial (RCT) was whether the incidence of implant failure could be reduced and whether radiological and clinical outcomes could be improved by using an additional anterior symphyseal plate in patients with class I obesity who have sustained Tile C1-2 and C1-3 pelvic ring injuries.

### Statistical analysis

The study analyzed data using SPSS version 20.0, with a sample size of 30 cases (15 per group) from a level I trauma center over 15 months, including 10% dropouts, to determine the frequency and percentage of vertically unstable open-book pelvic ring injuries.

In the study, parametric analysis was conducted using the t-test and Chi-square test for data analysis, maintaining a confidence interval of 95% and a margin of error of 5%. The researcher assumed that the data were normal according to the sample size (over 30) and the small standard deviation. The trial assumed that p-values of 0.05 or lower were sufficient to demonstrate statistical significance.

## Results

The study involved 36 patients (18 in each group) with a BMI ranging from 30 to 35; the mean age was 25.6 years for group A and 33 years for group B (Table 1). Group A included ten patients (55.6%) with Tile C1-2 pelvic ring injuries and eight patients (44.4%) with Tile C1-3 injuries. Group B included eight patients (44.4%) with Tile C1-2 injuries and ten patients (55.6%) with Tile C1-3 injuries (Tables 1 and 2). A Pfannenstiel incision for anterior ring fixation was utilized in all cases; after accurate reduction was achieved, posterior ring fixation proceeded via a single SI screw.

**Table 1:** Comparison between two methods regarding demographic data of patients

		Single superior plate (*N* = 18)				Double superior and anterior plates (*N* = 18)				t*	*P* value
		Min.	Max.	Mean	SD	Min.	Max.	Mean	SD		
Age (years)		18.00	60.00	25.61	10.55	18.00	60.00	33.06	13.27	1.86	0.07
		N		%		N		%		X^2**^	P value
Sex	Male	9		50.0%		12		66.7%		1.03	0.31
	Female	9		50.0%		6		33.3%			

**Table 2:** Comparison between two methods regarding pre-operative data

		Single superior plate (*N* = 18)		Double superior and anterior plates (*N* = 18)		X^2*^	P value
		N	%	N	%		
Side (according to posterior ring injury)	Right	10	55.6%	11	61.1%	0.11	0.74
	Left	8	44.4%	7	38.9%		
Classification of fracture	Tile C1-2	10	55.6%	8	44.4%	0.44	0.51
	Tile C1-3	8	44.4%	10	55.6%		

*Chi square test

The baseline demographic data including age and sex were included in our study as they have an implication either regarding bone quality and fixation techniques excluding osteoporotic and skeletally immature patients, and gender variations for the incidence of spermatic cord injury in males, that fortunately were not recorded in the study. Other demographic data including medical risk factors and previous surgical interventions, the authors confirm that all patients included in our study has no major medical risk factors that can have an impact on decision making, no previous surgical interventions in the abdomen including caesarian section that could be a confounding factor among the included sample, also patients with associated urogenital and visceral injuries were excluded. The investigators observed no significant difference in baseline demographic data between the two groups (Tables 1 and 2).

The authors analyzed the primary outcomes and found no significant difference in radiological assessment via Matta & Tornetta grading [13, 14] between the two groups (Table 3). The authors graded 66.7% of cases in Group A as excellent, 22.2% as good, and 11.1% as fair. Group B graded 66.7% of cases as excellent and 33.3% as good.

**Table 3:** Comparison between two methods regarding post-operative and follow up data

		Single superior plate (*N* = 18)				Double superior and anterior plates (*N* = 18)				t*	*P* value
		Min.	Max.	Mean	SD	Min.	Max.	Mean	SD		
Final Majeed score		68.00	79.00	76.39	3.01	58.00	79.00	73.78	6.41	1.56	0.13
Follow up (months)		12.00	18.00	13.39	1.97	12.00	18.00	13.17	1.62	0.37	0.71
		N		%		N		%		X^2**^	P value
Radiological assessment (by Matta & Tornetta radiological principles)	Excellent	12		66.7%		12		66.7%		2.05 FE	0.78
	Good	4		22.2%		6		33.3%			
	Fair	2		11.1%		0		0.0%			
Implant failure	No	17		94.4%		13		72.2%		3.20 FE	0.18
	Yes	1		5.6%		5		27.8%			
Wound infection	No	17		94.4%		13		72.2%		3.20 FE	0.18
	Yes	1		5.6%		5		27.8%			
Need for another operation	No	16		88.9%		10		55.6%		4.99	0.03
	Yes	2		11.1%		8		44.4%			
Final clinical assessment (by Majeed pelvic scoring system)	Excellent	11		61.1%		11		61.1%		4.49 FE	0.44
	Good	6		33.3%		2		11.1%			
	Fair	1		5.6%		4		22.2%			
	Poor	0		0.0%		1		5.6%			

Functional assessment via the Majeed score [15] was comparable between the two groups at one-year follow-up, with the mean score being 76.4 in group A and 73.8 in group B (Table 3); it was graded in group A: excellent in 61.1% of cases, good in 33.3% of cases, and fair in 5.6% of cases, while in group B: excellent in 61.1% of cases, good in 11.1% of cases, fair in 22.2% of cases, and poor in 5.6% of cases. The authors observed no statistically significant differences between the two groups (Table 3). Regarding implant failure, the results were statistically comparable in the two groups (Table 3), with higher rates in group B with anterior plate failure (5 cases, 27.8%), compared to group A with superior plate failure (1 case, 5.6%). (Table 3)

**Fig. 3:**
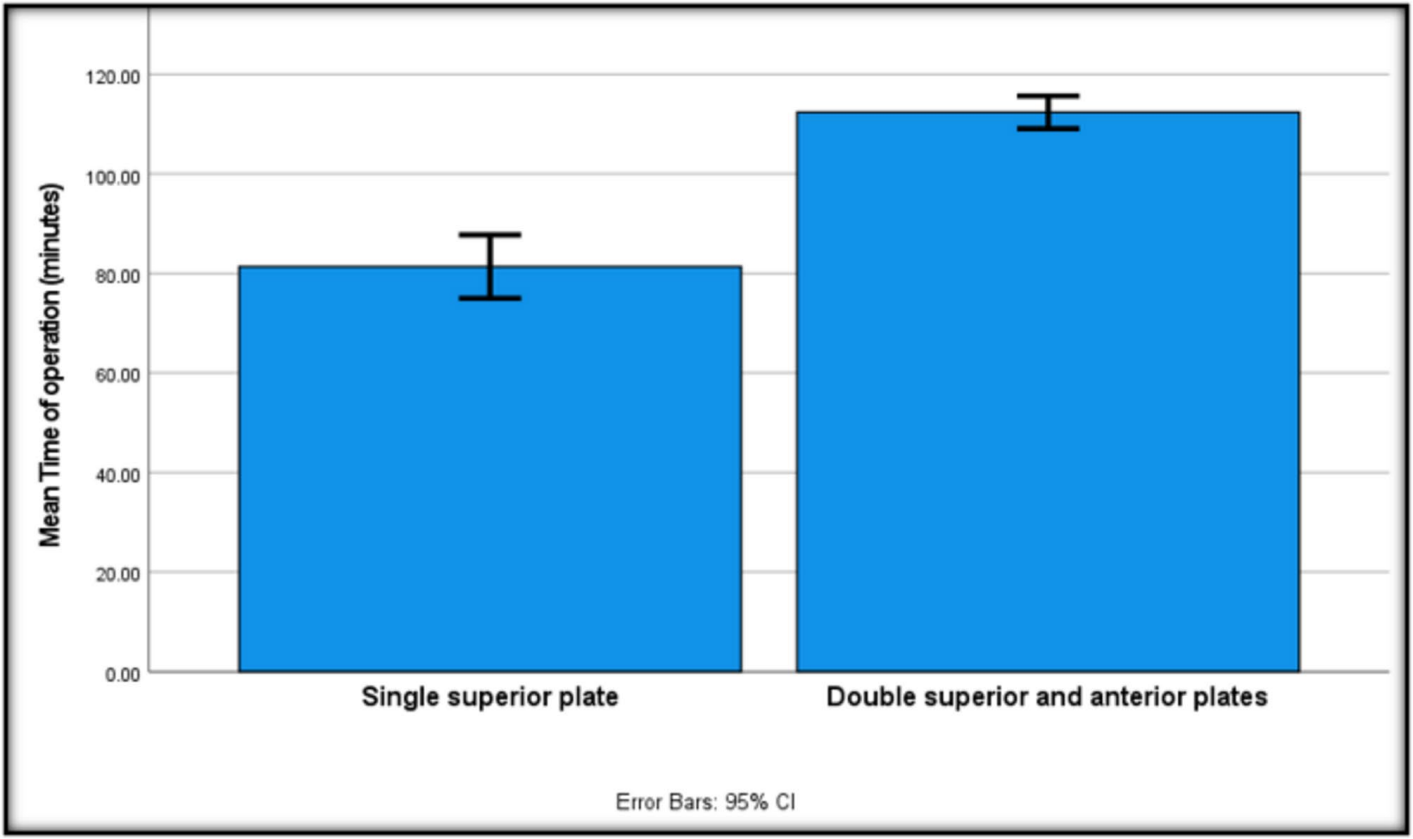
The error bar relationship between mean operation time in minutes and fixation techniques (HS)

As regards the secondary outcomes, the mean blood loss was comparable between the two groups, being slightly more in group B (422.2 ml) compared to group A (416.7 ml), with no statistically significant differences between the two groups (Table 4). The average surgical time was significantly lower (Fig. 3) in group A (81.4 min) than in group B (112.4 min) (Table 4). The Matta grading of intraoperative reduction revealed no statistically significant differences between the two groups, with group A graded as anatomical in 17 cases (94.4%) and acceptable in one case (5.6%). Group B was graded as anatomical in 14 cases (77.8%) and acceptable in four cases (22.2%) (Table 4).

**Table 4:** Comparison between two methods regarding operative data

		Single superior plate (*N* = 18)				Double superior and anterior plates (*N* = 18)				t*	*P* value
		Min.	Max.	Mean	SD	Min.	Max.	Mean	SD		
Time of operation (minutes)		70.00	110.00	81.39	12.81	100.00	120.00	112.39	6.58	9.13	< 0.001
Blood loss (ml)		350.00	500.00	416.67	42.01	350.00	500.00	422.22	35.24	0.43	0.67
		N		%		N		%		X^2**^	P value
Intraoperative assessment of reduction	Anatomical reduction	17		94.4%		14		77.8%		2.09 FE	0.34
	Acceptable reduction	1		5.6%		4		22.2%			

*Student t test **Chi square test (FE: Fisher Exact)

As regards wound infection, it occurred in one case in group A (5.6%) that required debridement once for superficial wound infection, without implant failure or need for revision of fixation. It occurred in five cases in group B (27.8%), out of which three cases required anterior plate removal for implant failure with debridement once, and two cases required only debridement once for superficial wound infection without implant failure. However, the two groups showed no statistically significant differences (Table 3). Finally, reoperation rates were significantly higher in group B (8 cases, 44.4%) compared to group A (2 cases, 11.1%). In group A, one case required debridement once, and the other required superior plate removal for implant failure. In group B, two cases required debridement once, two cases required anterior plate removal for implant failure, and three cases required anterior plate removal and debridement once (Table 3).

## Discussion

VS injuries, often caused by high-impact axial loading, refer to unstable Tile C injuries. Although some of the previous studies [18–20] reported no differences as regards implant failure between single versus double symphyseal plating in the management of such injuries, the broad inclusion criteria in these studies, which included all types of vertical shear injuries without excluding bilateral injuries or rami bony fractures, also neglecting the correlation between implant failure rates and higher BMI, raised concerns about the consistency of these results. In order to achieve statistically significant results, the authors chose narrow criteria for inclusion and standardized the posterior ring fixation method in people with class I obesity.

**Fig. 4:**
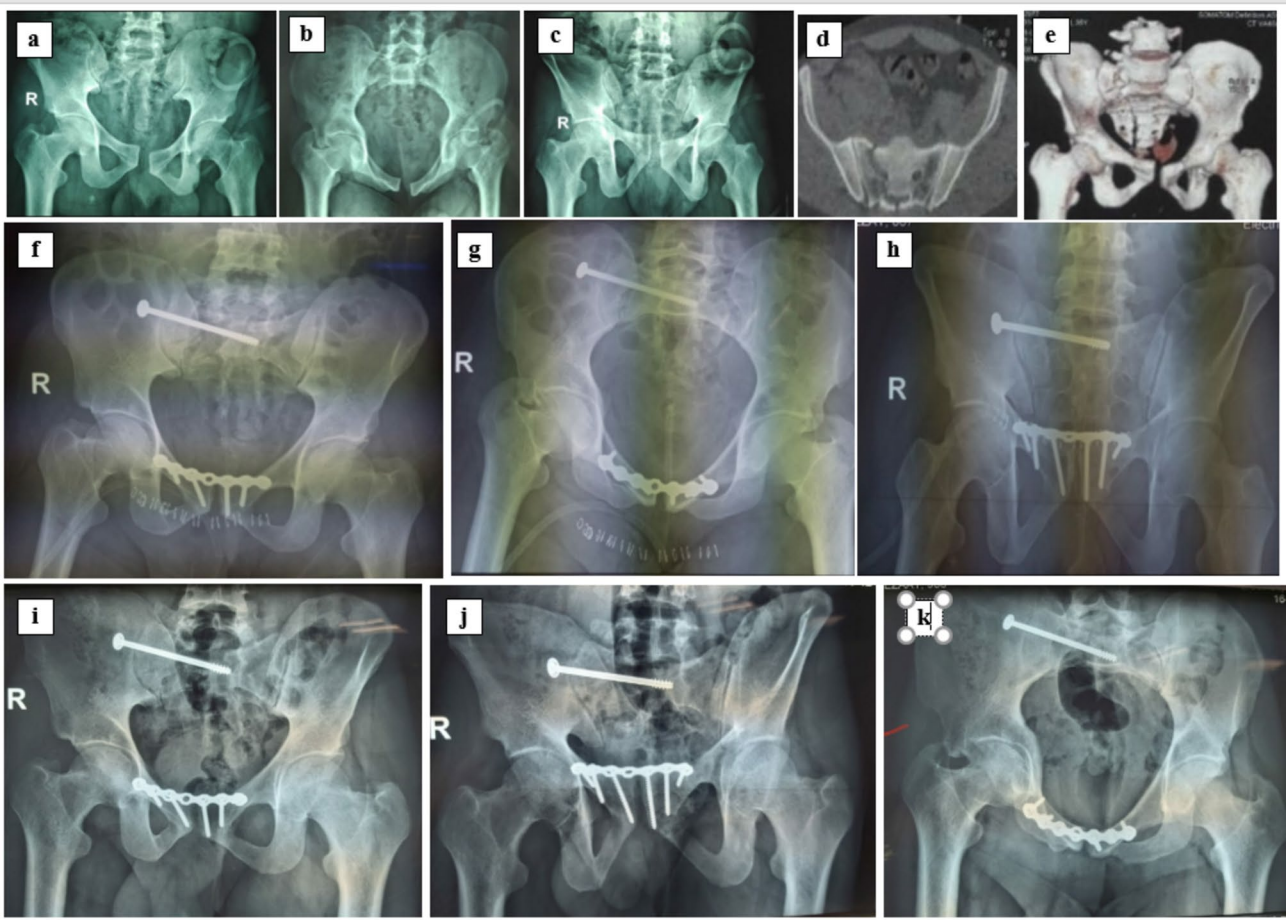
A 38-year-old male RTA. **a**-**e** preoperative radiographs and computed tomography (CT) scan showing Tile C1-3 injury (VS Fracture Sacrum). **F**-**H** postoperative radiographs show posterior ring fixation with a single SI screw and anterior ring fixation via a single superior symphyseal plate. **I**-**K** radiographs taken 14 months postoperatively

**Fig. 5:**
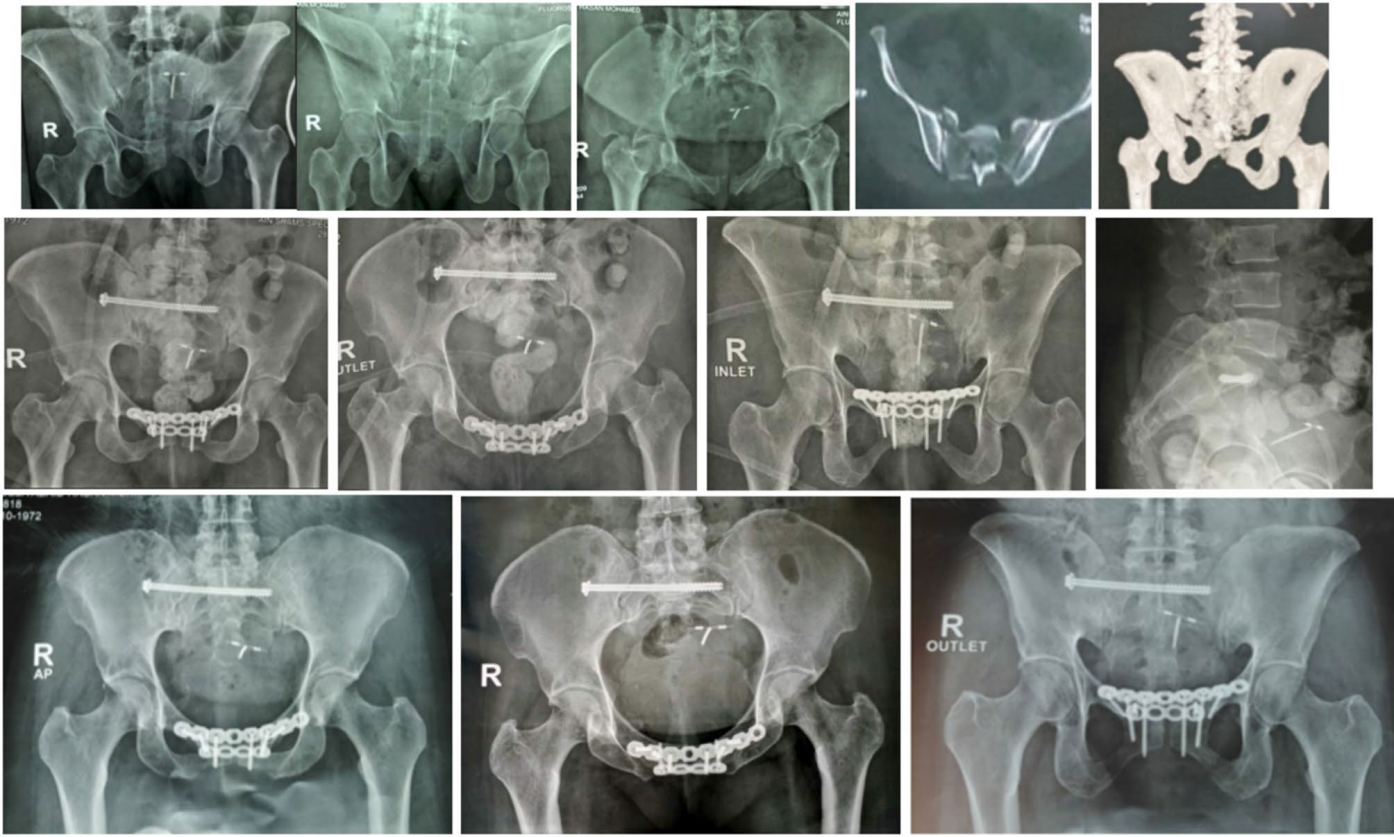
A 48-year-old female fell from a height. **a**-**e** preoperative radiographs and computed tomography (CT) scan showing Tile C1-3 injury (VS Fracture Sacrum); **f**-**i** postoperative radiographs showing posterior ring fixation with SI screw and anterior ring fixation via double superior and anterior symphyseal plates. **J**-**L** radiographs taken 15 months postoperatively

The study aimed to compare single superior plating and double superior and anterior plating in managing Tile C1-2 and C1-3 VS injuries in class I obesity patients. The results were comparable regarding the final radiological (Figs. 4 and 5) and functional outcomes, the mean clinical score, intra-operative reduction assessment, implant failure rates, mean blood loss, and the anterior ring fixation method. Double symphyseal plating in group B showed a longer operation time and higher rates of reoperations than single symphyseal plating in group A.

The randomized controlled trial included 36 patients, divided into two groups: group A, with 18 patients, received single symphyseal plating, while group B, also consisting of 18 patients, underwent double superior and anterior plating. The trial found no significant differences in demographic data between the two groups. Additionally, there were no significant differences in fracture patterns (*p* = 0.73) or in the final clinical (*p* = 0.44) and radiological outcomes (*p* = 0.78), mean clinical score (*p* = 0.13), intraoperative reduction assessment (*p* = 0.34), implant failure rates (*p* = 0.18), wound infection (*p* = 0.18), and average blood loss (*p* = 0.67) were similar in both groups. However, the single symphyseal plate in group A had a significantly shorter surgical duration (*p* < 0.001) and a less need for additional surgeries (*p* = 0.03), attributed to the less time needed to apply additional anterior plates in group A and the higher need for anterior plate removal and debridement for wound infection in group B.

Authors used Matta and Tornetta’s radiological principles [13, 14], and they found that the final radiological outcomes were similar for both fixation methods (*p* = 0.78). They graded group A as excellent in 67% of the cases, good in 22%, and fair in 11%, and they graded group B as excellent in 66.7% of the cases and good in 33.3%. The radiological outcomes were better than in the prospective study by Putnis et al. [21], with only 53% of patients (26 out of 49) graded as excellent or good. The incidence of implant failure according to Collinge et al. criteria [16, 17] was less than in the studies by Morris et al. [18] (28.8%), Tseng et al. [9] (32%), and Frietman et al. [19] (29.7%). It was higher in group B (5 cases, 27.8%) than in group A (one case, 5.6%); this explains the higher rates of reoperation in group B.

The final Majeed clinical outcome results were comparable to the retrospective systematic reviews by Vaidya et al. [3], Frietman et al. [19], Tseng K-Y et al. [9], and Baron et al. [22]. They compared single versus double symphyseal plating, or INFIX. 52, 37, 28, and 128 patients underwent these procedures, resulting in mean Majeed pelvic scores of 77.67, 76.3, 78.2, and 79, respectively. The authors analyzed the final functional outcomes in the current study using the Majeed pelvic scoring system [15] and found a correlation with the radiological outcome. Also, no statistically significant differences (*p* = 0.44) were noted between the two groups, with group A’s mean score being 76.4 and group B’s at 73.8 (Table 3). Group A graded the clinical outcomes excellent in 61% of cases, good in 33% of cases, and fair in 5.5% of cases, while Group B graded it excellent in 61% of cases, good in 11% of cases, fair in 22% of cases, and poor in 5.5% of cases.

In the current study, the wound infection rate in group A (5.6%) was lower than those reported by Rahman et al. (9%) [23] and Moussa et al. (14%) [24].; two prospective studies that utilized a posterior SI screw and a single anterior plate for management of Tile C injuries (20 and 22 patients, respectively). The results were comparable in both groups (*p* = 0.18), being higher in group B in 5 cases (27.8%) and one case in group A (5.6%). However, no statistically significant differences were observed among the two groups (see Table 4). Additionally, the intraoperative assessment of reduction via Matta grading [13, 14] yielded comparable results (*p* = 0.34) in both groups, with group A grading 17 cases (94.4%) as anatomical and 1 case (5.6%) as acceptable. While in group B, anatomical in 14 cases (77.8%) and acceptable in 4 cases (22.2%), the results in group A were slightly superior to the study by Moussa et al. [24]., with 90% of cases being graded as anatomical or acceptable. The mean intraoperative blood loss was nearly equal in the study (*p* = 0.67). However, Group A had a significantly shorter surgical time (81.4 min) compared to Group B (112.4 min), primarily due to the extra time required for anterior plate fixation. Also, group A showed statistically significant (*p* = 0.03) lower rates of reoperation (8 cases, 44.4%) compared to group B (2 cases, 11.1%), with a higher need for wound debridement or anterior plate removal in group B.

The study had limitations due to a small sample size, underpowered study with incomplete power analysis, and lack of long-term follow-up, primarily due to the narrow inclusion criteria of our patients and a rough estimate analysis of the sample size. Another limitation was the use of a single iliosacral screw for posterior ring fixation, although being biomechanically and clinically valid and evidence based in management of Tile C injuries, however more rigid fixation via two iliosacral screws allows earlier mobility and weight bearing while maintaining the reduction. A performance bias might have occurred since a single team conducted all operations. Consequently, later operations may have yielded improved results. The last limitation in the study was the lack of fracture displacement quantification for the amount of vertical instability which could limit the clinical context and implications, however, the study design was based on the direction of instability according to Tile classification without including a quantitative analysis of the amount of vertical displacement. Despite the previous limitations, this study was the first to compare such outcome measures in a prospective, randomized manner to investigate the potential benefits of using an additional anterior symphyseal plating through the same incision in Tile C1-2 and C1-3 injuries, aiming to achieve rigid fixation and reduce complications’ rate.

## Conclusion

The authors found that using a single high-quality symphyseal plate effectively addresses symphyseal diastasis in vertically unstable Tile C1-2 and C1-3 pelvic ring injuries among patients with class I obesity. This method lowers morbidity by reducing operation times and minimizing reoperation rates, while the inclusion of an additional anterior plate does not enhance the final radiological and clinical outcomes. In fact, it was associated with increased rates of implant failure and wound infection, even though they weren’t statistically significant but correlated with higher reoperation rates. The authors intend to refine the management protocol for Tile C injuries by conducting further level I trials, which will involve larger sample sizes and extended follow-up periods for the patients.

## Electronic supplementary material

Below is the link to the electronic supplementary material.

Supplementary Material 1

Supplementary Material 2

Supplementary Material 3

Supplementary Material 4

Supplementary Material 5

Supplementary Material 6

## Data Availability

No datasets were generated or analysed during the current study.

## Abbreviations

VS: Vertical shear
BMI: Body Mass Index
AP: Anteroposterior
INFIX: Anterior subcutaneous internal fixator
PR: Posterior ring
SI: Sacroiliac
RCT: Randomized clinical trial
PRI: Pelvic ring injuries
SD: Standard deviation
