# Supplementary material for: Single versus double symphyseal plating in management of tile C1-2 and C1-3 pelvic ring injuries: a randomized controlled trial
Source: BMC Surg. 2025 May 9;25:200. doi: 10.1186/s12893-025-02936-3 (PMC12063314; doi:10.1186/s12893-025-02936-3)
Supplement: Supplementary file 5 — Supplementary Material 5 [file 12893_2025_2936_MOESM5_ESM.docx]

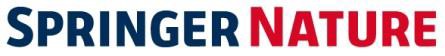


Disclosure of potential conflicts of interest

Authors disclose all relationships or interests that could have direct or potential influence or impart bias on the work. Although an author may not feel there is any conflict, disclosure of all relationships and interests provides a more complete and transparent process, leading to an accurate and objective assessment of the work. Awareness of real or perceived conflicts of interest is a perspective to which the readers are entitled. This is not meant to imply that a financial relationship with an organization that sponsored the research or compensation received for consultancy work is inappropriate.

Corresponding author of the manuscript submitted to [BMC musculoskeletal disorders] disclose any real or perceived conflict of interest. The corresponding author signs on behalf of all authors.

The corresponding author will include a statement in that reflects what is recorded in the potential conflict of interest disclosure form and prepared to send the potential conflict of interest disclosure form if requested during peer review or after publication on behalf of all authors (if applicable).

We have no potential conflict of interest.

| Category of disclosure | Description of Interest/Arrangement |
| --- | --- |
| Financial or non-financial interests | none |
| Conflicts of interest to declare that are relevant to the content of this article. | none |
| Affiliations with or involvement in any organization or entity with any financial interest or non-financial interest in the subject matter or materials discussed in this manuscript. | none |
| Financial or proprietary interests in any material discussed in this article | none |

Article title **Single Versus Double Symphyseal Plating in Management of Vertically Unstable Tile C1-2 and C1-3 Pelvic Ring Injuries: A Randomized Controlled Trial**

Corresponding author name: Islam Sayed Moussa

Herewith I confirm, on behalf of all authors, that the information provided is accurate.

Author signature: Islam moussa

Date: 31/10/2024
