# Supplementary material for: Single versus double symphyseal plating in management of tile C1-2 and C1-3 pelvic ring injuries: a randomized controlled trial
Source: BMC Surg. 2025 May 9;25:200. doi: 10.1186/s12893-025-02936-3 (PMC12063314; doi:10.1186/s12893-025-02936-3)
Supplement: Supplementary file 6 — Supplementary Material 6 [file 12893_2025_2936_MOESM6_ESM.doc]

**Single Versus Double Symphyseal Plating in Management of Vertically Unstable Tile C1-2 and C1-3 Pelvic Ring Injuries: A Randomized Controlled Trial**

**Islam Sayed Moussa * ^1^, Ibrahim Mahmoud Abdelmonem ^1^, Amr Mohammed Nagy ^1^**

Department of Orthopedics and Traumatology, Faculty of Medicine, Ain Shams University

56 Ramses Street, Abbasia, Cairo11522, Egypt

Corresponding author: Islam S.Moussa *

e-mail: [Islammoussa@med.asu.edu.eg](mailto:Islammoussa@med.asu.edu.eg) *

ORCID of the corresponding author: 0000-0003-2685-2765

Tel: + 00201004950774; Fax: + 24178206
